# High serum amyloid A predicts risk of cognitive impairment after lacunar infarction: Development and validation of a nomogram

**DOI:** 10.3389/fneur.2022.972771

**Published:** 2022-08-24

**Authors:** Sheng Ye, Huiqing Pan, Weijia Li, Bing Wang, Jingjing Xing, Li Xu

**Affiliations:** ^1^Department of Emergency, The Second Affiliated Hospital of Wannan Medical College, Wuhu, China; ^2^School of Clinical Medicine, Wannan Medical College, Wuhu, China; ^3^Department of Neurology, The Second Affiliated Hospital of Wannan Medical College, Wuhu, China

**Keywords:** serum amyloid A, cognitive impairment, lacunar infarction, nomogram, prediction model

## Abstract

**Background:**

Post-stroke cognitive impairment (PSCI) after lacunar infarction was worth attention in recent years. An easy-to-use score model to predict the risk of PSCI was rare. This study aimed to explore the association between serum amyloid A (SAA) and cognitive impairment, and it also developed a nomogram for predicting the risk of PSCI in lacunar infarction patients.

**Methods:**

A total of 313 patients with lacunar infarction were enrolled in this retrospective study between January 2021 and December 2021. They were divided into a training set and a validation set at 70%:30% randomly. The Chinese version of the Mini-Mental State Examination (MMSE) was performed to identify cognitive impairment 3 months after discharge. Univariate and multivariate logistic regression analyses were used to determine the independent risk factors for PSCI in the training set. A nomogram was developed based on the five variables, and the calibration curve and the receiver operating characteristic (ROC) curve were drawn to assess the predictive ability of the nomogram between the training set and the validation set. The decision curve analysis (DCA) was also conducted in both sets.

**Results:**

In total, 52/313 (16.61%) participants were identified with PSCI. The SAA levels in patients with PSCI were significantly higher than non-PSCI patients in the training set (*P* < 0.001). After multivariate analysis, age, diabetes mellitus, white blood count, cystatin C, and SAA were independent risk predictors of PSCI. The nomogram demonstrated a good discrimination performance between the training set (AUC = 0.860) and the validation set (AUC = 0.811). The DCA showed that the nomogram had a well clinical utility in the two sets.

**Conclusion:**

The increased SAA is associated with PSCI in lacunar infarction patients, and the nomogram developed with SAA can increase prognostic information for the early detection of PSCI.

## Introduction

Stroke is the second leading cause of death around the world, which endangers the life quality and safety of patients due to high morbidity and high disability (1). Especially, post-stroke cognitive impairment (PSCI) is the most common critical issue concerning population health and the burden on caregivers in an aging society (2, 3). The survivors of stroke have an increased risk of progressive cognitive impairment, even minor stroke (4). The presence of PSCI also affects the treatment of stroke patients in turn and nearly increases two-fold the risk of adverse outcomes (5). Lacunar infarction accounts for about 25% of stroke patients, and approximately half of the patients develop cognitive impairment in subsequent years (6). With the increased duration of ischemia and decreased mortality with minor stroke, the number of patients with PSCI will be increased (7). Therefore, it is crucial to realize the associations between PSCI and predictive factors, especially in high-risk patients.

The available evidence confirms that hypertension, diabetes, smoking, and other vascular risk factors are highly correlated with the increased risks of PSCI (8). Besides, frontal lobe dysfunction and brain gray matter atrophy were also associated with cognitive impairment in lacunar patients (9). Recently, the developed SIGNAL2 scale and CHANGE scale based on the clinical characteristics and neuroimaging variables were useful to identify PSCI after stroke (10, 11). However, the scale depends on the neurologist's appraising of the MRI and was difficult to promote in the community. In addition, the expression of biomarkers, such as interleukin 6 (IL-6), C-reactive protein (CRP), serum uric acid (UA), and malondialdehyde (MDA), was also independently associated with PSCI in increasing studies (12, 13). Therefore, it is necessary to explore reliable biomarkers to identify patients at higher risk of PSCI easily and conveniently.

Serum amyloid A (SAA) protein is a protein of only 104 amino acids and is mainly synthesized in the liver (14). As an acute phase protein (APP), SAA was significantly upregulated in acute and chronic inflammatory conditions (such as trauma, infection, and ischemia), which was in response to the elevator of the inflammatory cytokines IL-6 and tumor necrosis factor (TNF)-α during the acute-phase response (15). Schweizer et al.'s study (16) found that SAA was a novel blood biomarker, which was independent to predict post-stroke infection among ischemic stroke patients. A recent study confirmed that the increased secretion of SAA could activate the inflammatory response of microglia and stimulate NLRP3 activation in microglia after stroke, which induced neurological inflammation (17). Although studies have indicated that elevated SAA was associated with short-term cognitive impairment after ischemic stroke (18), the role of SAA has not yet been evaluated in the cognitive impairment after lacunar infarction.

Therefore, we aimed to develop and verify a nomogram to predict the risk of PSCI in lacunar infarction patients, which will be convenient for clinicians to identify cognitive disorders early and conveniently.

## Methods

### Study design and patients

This study retrospectively enrolled patients with lacunar infarction who were hospitalized at the Second Affiliated Hospital of Wannan Medical College between January 2021 and December 2021. All patients were admitted to the hospital within 7 days of symptom onset with a National Institute of Health Stroke Scale (NIHSS) score ≤3. This study was approved by the Institutional Review Board of the Second Affiliated Hospital of Wannan Medical College (No. WYEFYLS202205) and conducted by the guiding principles of the Declaration of Helsinki.

The inclusion criteria were as follows: (1) age >18 years; (2) patients who met the diagnostic criteria for lacunar infarction confirmed on cranial computed tomography (CT) scan or magnetic resonance imaging (MRI) examination; and (3) patients who were able to complete scale measurements. The patients were excluded if they had any of the following: (1) previous diagnosis with dementia or Alzheimer's disease; (2) cardioembolic source or large-vessel diseases (large artery stenosis >50%); (3) patients with incomplete clinical data; (4) had been treated with intervention and thrombolytic therapy; and (5) loss to follow-up.

**Figure 1:**
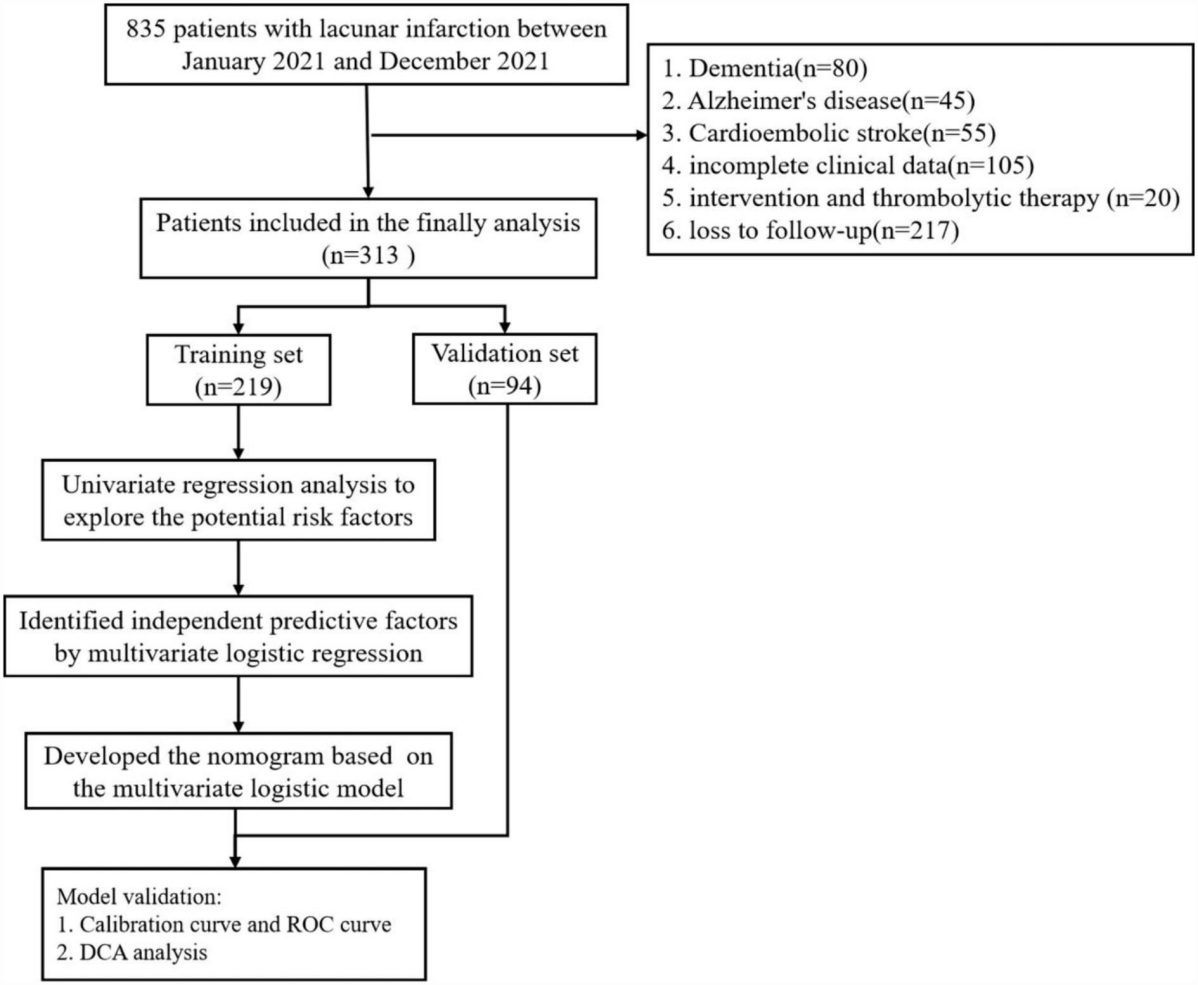
Schematic of patient's inclusion process and flowchart with the study.

Finally, a total of 313 patients were enrolled in this study and were randomly divided into a training set and a validation set at 70%:30% (Figure 1).

### Baseline clinical characteristics collection

The baseline clinical characteristics were collected within 24 h of admission from the health information system (HIS). The first part was demographic characteristics, such as age, gender, education time, initial National Institutes of Health Stroke Scale (NIHSS) score, and vital signs (blood pressure, heart rate, temperature, breath rate). The second part was comorbidities (diabetes mellitus, atrial fibrillation, coronary heart disease, hypertension, tumor, and chronic obstructive pulmonary disease). The third part was laboratory examinations, which included red blood count, white blood count, hemoglobin, platelet, cystatin C, and total cholesterol.

### Serum amyloid A

All blood samples were collected in the morning from all patients within 24 h of admission, and all patients were fasting for more than 8 h. The blood samples were collected with heparin anticoagulant tubes and centrifuged at 1,000 *g* for 5 min to separate serum. The serum amyloid A level was measured by the latex-enhanced immunoturbidimetric method with the automatic biochemical analyzer (Hitachi-7600). The reference value of serum amyloid A ranged from 0 to 10.00 mg/L. All sample testing was performed by laboratory personnel blinded to the study.

### Lacunar infarction definition

All patients routinely completed MRI examination or CT scanning. Lacunar infarction was defined as lacunar infarcts <20 mm in the subcortical or brain stem verified by CT or MRI (19, 20). Radiologic images were read by a radiologist and were reviewed by an experienced neurologist.

### Assessment of cognitive function

Cognitive function was evaluated by experienced neurological physicians using the Chinese version of the Mini-Mental State Examination (MMSE) scale 3 months after lacunar infarction (21). The MMSE scale was widely used in cognitive function assessment in China (22, 23). The total score of MMSE was 30, and the lower scores indicated the worse cognitive function. According to the previous studies, a score of MMSE <24 was considered as a cognitive impairment in this study (24, 25).

### Statistical analysis

The statistical analysis was performed by using SPSS 25.0 and R software (version 3.6.2). Continuous variables with normal distribution were expressed as mean ± standard deviation, and the interquartile range were not normally distributed. Categorical variables were presented as frequency (percentage).

Univariate analysis was applied to screen the potential risk factors for PSCI. To determine independent risk factors for PSCI in lacunar infarction patients, the variables with *P* < 0.05 in the univariate analysis were included in the multivariate logistic regression model. Then, the predictive nomogram was developed based on the independent risk factors by using the “rms” package in R software. The receiver operating characteristic (ROC) curve was generated, and the area under the curve (AUC) of ROC was used to evaluate the discrimination of the nomogram. Meanwhile, the AUC of the nomogram was compared with all the independent risk variables. Finally, the net benefit of the decision curve analysis (DCA) was drawn to estimate the clinical value of the nomogram in the training set and validation set, respectively. Linear regression analysis was used to analyze the association between the serum SAA and the MMSE score. The statistical significance for all variables was set at *P* < 0.05 (two-sided tests), and the regression coefficients reported 95% confidence intervals (CI).

## Results

### Clinical characteristics of patients in the training and validation set

A total of 313 patients with lacunar infarction were included in this study, of which 219 patients were in the training set and 94 patients were in the validation set; 38 (17.40%) patients in the training set and 14 (14.90%) patients in the validation set were diagnosed with PSCI, respectively. The characteristics of patients in the training set and the validation set were no significant differences in Table 1 (*P* > 0.05).

**Table 1:** Baseline and clinical characteristics of lacunar infarction patients in the training set and validation set.

**Variables**	**Total (*n* = 313)**	**Training set (*n* = 219)**	**Validation set (*n* = 94)**	***t*/*z*/χ^2^**	***P*-value**
**Demographic characteristics**
Age, years, Mean ± SD	69.57 ± 10.83	69.62 ± 10.81	69.45 ± 10.95	0.127	0.899
Male, *n* (%)	153 (48.90)	102 (46.60)	51 (54.30)	1.552	0.213
Education, years, median (IQR)	5.00 (3.00, 6.00)	5.00 (3.00, 6.00)	5.00 (3.00, 8.00)	−0.955	0.340
**Comorbidity**
Diabetes mellitus, *n* (%)	65 (20.80)	45 (20.50)	20 (21.30)	0.021	0.884
Atrial fibrillation, *n* (%)	43 (13.70)	25 (11.40)	18 (19.10)	3.319	0.068
Coronary heart disease, *n* (%)	53 (16.90)	41 (18.70)	12 (12.80)	1.658	0.198
Hypertension, *n* (%)	153 (48.90)	113 (51.60)	40 (42.60)	2.153	0.142
COPD, *n* (%)	42 (13.40)	33 (15.10)	9 (9.60)	1.709	0.191
Tumor, *n* (%)	33 (10.50)	25 (11.40)	8 (8.50)	0.588	0.443
**Laboratory examination**
WBC, × 10^9^/L, median (IQR)	5.47 (4.52, 6.80)	5.46 (4.54, 6.91)	5.49 (4.47, 6.46)	−1.155	0.248
RBC, × 10^12^/L, median (IQR)	4.20 (3.82, 4.55)	4.19 (3.81, 4.56)	4.23 (3.90, 4.54)	−0.306	0.760
HB, g/L, median (IQR)	127.00 (115.00, 139.00)	127.00 (114.00, 139.00)	127.00 (117.00, 140.00)	−0.539	0.590
PLT, × 10^12^/L, median (IQR)	164.00 (135.00, 212.00)	166.00 (140.00, 208.00)	159.50 (123.75, 216.50)	−1.292	0.196
PT, s, median (IQR)	11.00 (10.40, 12.05)	10.90 (10.40, 12.10)	11.10 (10.50, 11.93)	−0.399	0.690
APTT, s, median (IQR)	25.30 (22.80, 28.60)	25.00 (22.80, 28.40)	25.50 (22.80, 28.78)	−0.625	0.532
FIB, g/L, median (IQR)	2.40 (2.10, 2.96)	2.50 (2.10, 3.00)	2.30 (2.00, 2.90)	−1.315	0.188
CysC, mg/L, median (IQR)	1.03 (0.87, 1.23)	1.03 (0.87, 1.23)	1.02 (0.87,1.22)	−0.591	0.555
ApoA1, g/L, median (IQR)	1.32 (1.12, 1.59)	1.33 (1.12, 1.59)	1.30 (1.11,1.55)	−0.559	0.576
ApoB, g/L, median (IQR)	0.83 (0.66, 1.02)	0.81 (0.63, 1.03)	0.88 (0.71, 1.02)	−1.292	0.196
SAA, mg/L, median (IQR)	9.00 (4.50, 19.50)	9.30 (4.80, 21.10)	8.10 (3.75, 17.20)	−1.249	0.212
TC, mmol/L, median (IQR)	4.09 (3.40, 4.87)	4.09 (3.39, 4.84)	4.09 (3.39, 4.96)	−0.290	0.772
LDL, mmol/L, median (IQR)	2.15 (1.51, 22.74)	2.12 (1.49, 2.71)	2.19 (1.51, 2.76)	−0.458	0.647
TG, mmol/L, median (IQR)	1.16 (0.83, 1.64)	1.18 (0.84, 1.62)	1.14 (0.74, 1.67)	−0.940	0.347
ALT, μ/L, median (IQR)	14.00 (10.00, 21.00)	15.00 (10.00, 21.00)	14.00 (9.75, 20.25)	−0.472	0.637
AST, μ/L, median (IQR)	22.00 (18.00, 27.00)	22.00 (18.00, 27.00)	22.00 (17.00, 27.00)	−0.450	0.653
ALB, g/L, median (IQR)	40.30 (37.60, 43.20)	40.10 (37.30, 43.00)	40.70 (38.30, 43.33)	−1.145	0.252
GLO, g/L, median (IQR)	28.10 (25.10, 30.95)	28.10 (25.10, 30.80)	27.85 (24.73, 31.40)	−0.292	0.771
TBIL, μmol/L, median (IQR)	11.70 (9.20, 14.95)	11.70 (9.10, 14.80)	11.65 (9.75, 16.20)	−0.287	0.774
GLU, mmol/L, median (IQR)	5.52 (4.77, 7.22)	5.61 (4.79, 7.54)	5.40 (4.64, 6.88)	−1.563	0.118
CREA, μmol/L, median (IQR)	73.00 (62.00, 87.30)	72.00 (62.00, 88.00)	74.90 (63.00, 86.25)	−0.401	0.688
CK, mmol/L, median (IQR)	77.00 (55.50, 109.50)	81.00 (58.00, 117.00)	71.50 (52.75, 94.50)	−1.839	0.066
UA, mmol/L, median (IQR)	332.80 (275.00, 402.00)	330.00 (275.00, 392.30)	344.00 (275.25, 418.25)	−1.046	0.296
MMSE, score, median (IQR)	26.00 (25.00, 28.00)	26.00 (25.00, 28.00)	27.00 (25.00, 28.00)	−1.611	0.107
**Subtypes of lacunar infarction**				6.418	0.268
Pure motor hemiparesis, *n* (%)	47 (15.00)	36 (16.40)	11 (11.70)		
Pure sensory stroke, *n* (%)	83 (26.50)	64 (29.20)	19 (20.20)		
Sensorimotor syndrome, *n* (%)	55 (17.60)	38 (17.40)	17 (18.10)		
Ataxic hemiparesis, *n* (%)	40 (12.80)	27 (12.30)	13 (13.80)		
Dysarthria clumsy, *n* (%)	36 (11.50)	23 (10.50)	13 (13.80)		
Atypical lacunar syndromes, *n* (%)	52 (16.60)	31 (14.20)	21 (22.30)		

SD, standard deviation; IQR, interquartile range; COPD, chronic obstructive pulmonary disease; RBC, red blood count; WBC, white blood count; HB, hemoglobin; PLT, platelet; CysC, cystatin C; TC, total cholesterol; LDL, low-density lipoprotein; TG, triglyceride; ALB, albumin; TBIL, total bilirubin; CREA, creatinine; BUN, blood urea nitrogen; UA, uric acid; ALT, alanine transaminase; SAA, serum amyloid A; MMSE, Mini-Mental State Examination.

### Baseline characteristics of patients stratified by PSCI in the training set

Descriptive analysis revealed that significant differences between the two groups were confirmed for age (*P* < 0.001), years of education (*P* = 0.001), diabetes mellitus (*P* = 0.006), white blood count (*P* < 0.001), APTT (*P* = 0.036), cystatin C (*P* < 0.001), ApoA1 (*P* = 0.005), and serum amyloid A (*P* < 0.001) in Table 2.

**Table 2:** Baseline and clinical characteristics of lacunar infarction patients in the training set.

**Variables**	**Total (*n* = 219)**	**PSCI (*n* = 38)**	**Non-PSCI (*n* = 181)**	***t*/*z*/χ^2^**	***P*-value**
**Demographic characteristics**
Age, years, Mean ± SD	69.62 ± 10.81	76.47 ± 10.07	68.18 ± 10.42	4.486	<0.001
Male, *n* (%)	102 (46.60)	16 (42.10)	86 (47.50)	0.369	0.543
Education, years, median (IQR)	5.00 (3.00, 6.00)	3 (0.00, 5.00)	5.00 (3.00, 6.00)	−3.350	0.001
**Comorbidity**
Diabetes mellitus, *n* (%)	45 (20.50)	14 (36.80)	31 (17.10)	7.477	0.006
Atrial fibrillation, *n* (%)	25 (11.40)	7 (18.40)	18 (9.90)	2.231	0.135
Coronary heart disease, *n* (%)	41 (18.70)	10 (26.30)	31 (17.10)	1.743	0.187
Hypertension, *n* (%)	113 (51.60)	25 (65.80)	88 (48.60)	3.708	0.054
COPD, *n* (%)	33 (15.10)	7 (18.40)	26 (14.40)	0.404	0.525
Tumor, *n* (%)	25 (11.40)	3 (7.90)	22 (12.20)	0.564	0.453
**Laboratory examination**
WBC, × 10^9^/L, median (IQR)	5.46 (4.54, 6.91)	6.97 (5.43, 8.16)	5.29 (4.47, 6.63)	−3.705	<0.001
RBC, × 10^12^/L, median (IQR)	4.19 (3.81, 4.56)	4.12 (3.66, 4.43)	4.20 (3.82, 4.57)	−0.941	0.347
HB, g/L, median (IQR)	127.00 (114.00, 139.00)	118.00 (106.50, 137.50)	128.00 (116.50, 139.00)	−1.559	0.119
PLT, × 10^12^/L, median (IQR)	166.00 (140.00, 208.00)	191.50 (143.50, 249.25)	164.00 (138.50, 200.00)	−1.507	0.132
PT, s, median (IQR)	10.90 (10.40, 12.10)	11.40 (10.50, 12.98)	10.90 (10.40, 12.05)	−1.754	0.079
APTT, s, median (IQR)	25.00 (22.80, 28.40)	26.45 (23.85, 32.28)	24.70 (22.80, 28.05)	−2.098	0.036
FIB, g/L, median (IQR)	2.50 (2.10, 3.00)	2.70 (2.15, 3.03)	2.40 (2.08, 2.99)	−1.851	0.064
CysC, mg/L, median (IQR)	1.03 (0.87, 1.23)	1.24 (1.03, 1.58)	1.01 (0.86, 1.17)	−4.089	<0.001
ApoA1, g/L, median (IQR)	1.33 (1.12, 1.59)	1.18 (1.06, 1.42)	1.37 (1.14, 1.61)	−2.783	0.005
ApoB, g/L, median (IQR)	0.81 (0.63, 1.03)	0.79 (0.64, 1.00)	0.83 (0.63, 1.04)	−0.542	0.588
SAA, mg/L, median (IQR)	9.30 (4.80, 21.10)	29.55 (10.15, 48.18)	8.40 (4.00, 15.10)	−5.031	<0.001
TC, mmol/L, median (IQR)	4.09 (3.39, 4.84)	4.08 (3.56, 4.78)	4.09 (3.34, 4.85)	−0.420	0.675
LDL, mmol/L, median (IQR)	2.12 (1.49, 2.71)	2.35 (1.88, 2.87)	2.09 (1.46, 2.66)	−1.878	0.060
TG, mmol/L, median (IQR)	1.18 (0.84, 1.62)	1.12 (0.87, 1.35)	1.20 (0.83, 1.67)	−0.396	0.692
ALT, μ/L, median (IQR)	15.00 (10.00, 21.00)	14.00 (8.00, 21.50)	15.00 (10.00, 21.00)	−0.486	0.627
AST, μ/L, median (IQR)	22.00 (18.00, 27.00)	22.00 (18.75, 26.00)	22.00 (18.00, 27.50)	−0.059	0.953
ALB, g/L, median (IQR)	40.10 (37.30, 43.00)	39.25 (36.38, 41.78)	40.20 (37.45, 43.20)	−1.364	0.172
GLO, g/L, median (IQR)	28.10 (25.10, 30.80)	29.25 (25.10, 32.78)	27.90 (25.15, 30.45)	−1.266	0.206
TBIL, μmol/L, median (IQR)	11.70 (9.10, 14.80)	12.45 (9.08, 14.95)	11.60 (9.05, 14.80)	−0.504	0.614
GLU, mmol/L, median (IQR)	5.61 (4.79, 7.54)	6.32 (4.92, 9.19)	5.47 (4.77, 7.25)	−1.705	0.088
CREA, μmol/L, median (IQR)	72.00 (62.00, 88.00)	79.35 (66.60, 89.00)	70.00 (61.00, 86.00)	−1.960	0.050
CK, mmol/L, median (IQR)	81.00 (58.00, 117.00)	73.00 (54.75, 115.75)	81.00 (58.00, 117.50)	−0.752	0.452
UA, mmol/L, median (IQR)	330.00 (275.00, 392.30)	360.00 (286.75, 400.10)	322.00 (266.50, 390.85)	−1.677	0.094

PSCI, post-stroke cognitive impairment; SD, standard deviation; IQR, interquartile range; COPD, chronic obstructive pulmonary disease; RBC, red blood count; WBC, white blood count; HB, hemoglobin; PLT, platelet; CysC, cystatin C; TC, total cholesterol; LDL, low-density lipoprotein; TG, triglyceride; ALB, albumin; TBIL, total bilirubin; CREA, creatinine; BUN, blood urea nitrogen; UA, uric acid; ALT, alanine transaminase; SAA, serum amyloid A.

### Identifying the independent risk factors for PSCI

All the potential risk factors (*P* < 0.05) in the univariate regression analysis were included in the multivariate regression model. Multivariate logistic regression analysis revealed that age (OR = 1.099, 95%CI: 1.012–1.193, *P* = 0.025), diabetes mellitus (OR = 2.679, 95% CI: 1.029–6.976, *P* = 0.044), white blood count (OR = 1.271, 95% CI: 1.028–1.572, *P* = 0.027), cystatin C (OR = 3.118, 95% CI:1.053–9.228, *P* = 0.040), and serum amyloid A (OR = 1.031, 95% CI: 1.009–1.054, *P* = 0.007) were independent risk predictors of PSCI in patients with lacunar infarction (Table 3).

**Table 3:** Univariate and multivariate logistic regression analyses of risk factors for PSCI in the training set.

**Variables**	**Univariate logistic regression**			**Multivariate logistic regression**		
	***β***	**Odds ratio (95% CI)**	***P*-value**	***β***	**Odds ratio (95% CI)**	***P*-value**
Age, years	1.092	1.047–1.138	<0.001	1.099	1.012–1.193	0.025
Education, years	0.828	0.737–0.930	0.001	1.098	0.863–1.396	0.446
Diabetes mellitus						
No		Ref			Ref	
Yes	2.823	1.315–6.061	0.008	2.679	1.029–6.976	0.044
WBC	1.440	1.195–1.736	<0.001	1.271	1.028–1.572	0.027
APTT	1.047	1.003–1.092	0.036	1.031	0.985–1.080	0.194
CysC	4.569	1.857–11.245	0.001	3.118	1.053–9.228	0.040
ApoA1	0.183	0.056–0.599	0.005	0.593	0.140–2.518	0.479
SAA	1.044	1.025–1.064	<0.001	1.031	1.009–1.054	0.007

WBC, white blood count; CysC, cystatin C; SAA, serum amyloid A; APTT, activated partial thromboplastin time.

### The predictive nomogram development

**Figure 2:**
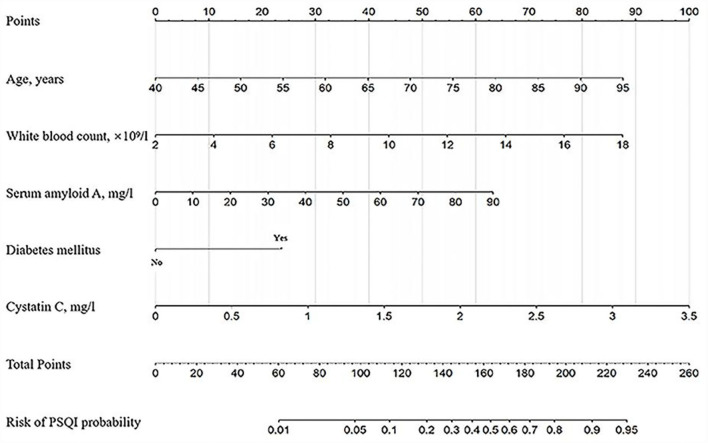
The nomogram for predicting the risk of PSCI probability based on the 5 independent prognostic factors. For example, a 65-year-old (40 points) patient with diabetes mellitus (25 points) with white blood counts of 12.0 × 10^9^/L (55 points), serum amyloid A of 50 mg/L (35 points), and cystatin C of 1.5 mg/L (42 points) arrived at a total point of 197, with a probability of 80% to develop PSCI.

The nomogram was developed for predicting the risk of PSCI probability based on the results from the multivariate logistic model, which included five variables (Figure 2). A vertical line was drawn up to the “Point” axis to calculate the score of each variable, and the total score was summarized by the preliminary scores. The total score was located on the “Total Points” axis, and then, the predicted risk of PSCI probability could be located on the bottom axis.

### The performance of the nomogram

**Figure 3:**
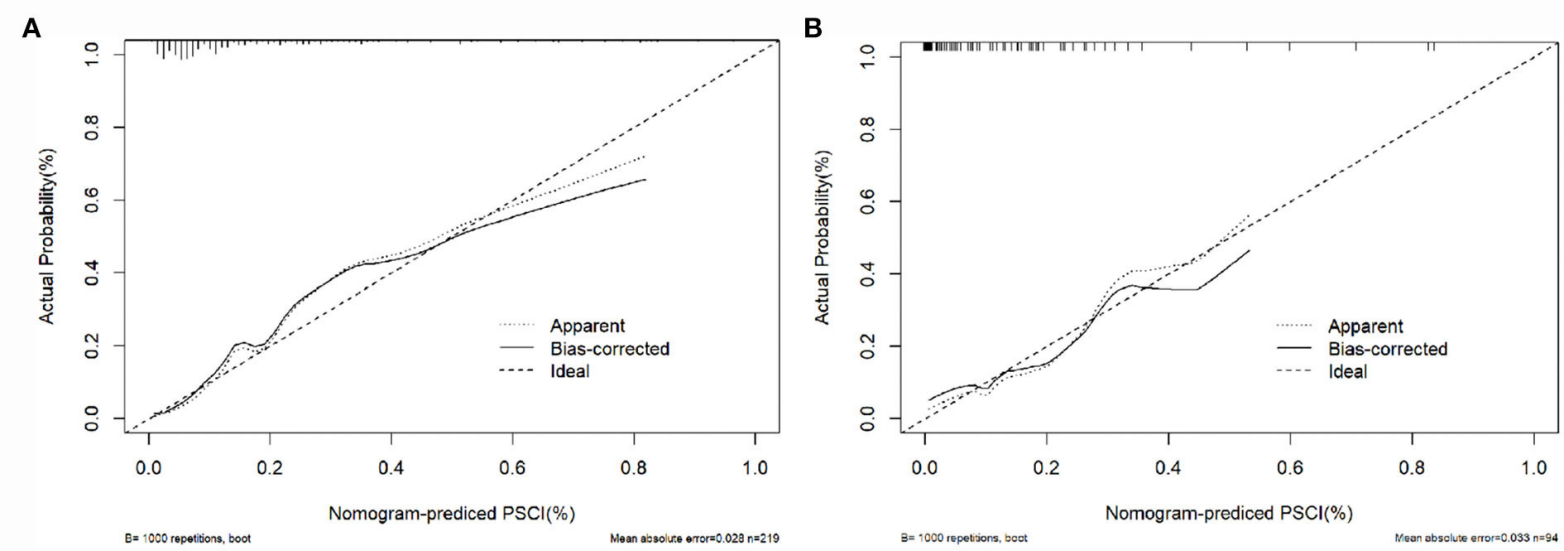
Calibration curves of the nomogram in the training set and the validation set. **(A)** The nomogram in the training set (*n* = 219); **(B)** the nomogram in the validation set (*n* = 94). The *y*-axis represents the observed rate of PSCI, and the *x*-axis represents the nomogram-predicted probability of PSCI. The dotted lines represented by the nomogram are closer to the diagonal gray lines representing a better prediction.

The calibration curve of the nomogram for the probability of PSCI demonstrated a good agreement between prediction and observation for both sets (Figure 3). The Hosmer-Lemeshow H test indicated that the model did not depart from perfect fit, which had non-statistical significance in the training set (*P* = 0.336) and validation set (*P* = 0.399).

**Figure 4:**
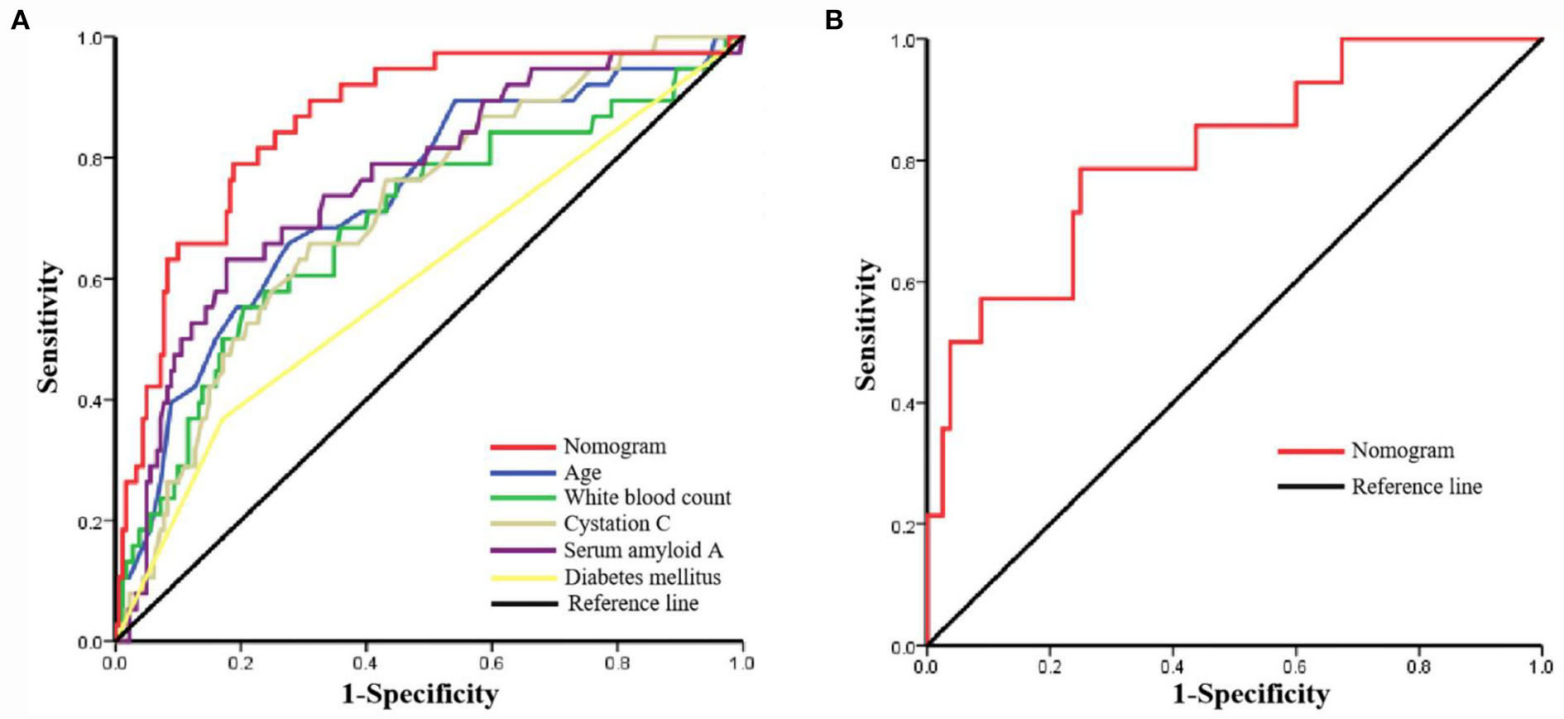
The receiver operating characteristic (ROC) curve of the nomogram in the training set and the validation set. **(A)** ROC in the training set; **(B)** ROC in the validation set.

The AUC for the nomogram was 0.860 (95% CI: 0.794–0.925) in the training set (Figure 4A) and was confirmed to be 0.811 (95% CI: 0.686–0.936) through internal validation in the validation set (Figure 4B), which demonstrated that the nomogram had a greater discriminatory performance. In addition, the discrimination ability of the nomogram calculated by the AUC was superior to the other risk factors in the training set: age (0.734, 95%CI: 0.643–0.824, *P* < 0.001), diabetes mellitus (0.599, 95% CI: 0.493–0.704, *P* = 0.056), white blood count (0.691, 95% CI: 0.592–0.791, *P* < 0.001), cystatin C (0.711, 95% CI: 0.625–0.797, *P* < 0.001), and serum amyloid A (0.760, 95% CI: 0.674–0.846, *P* < 0.001; Figure 4A).

### Clinical use

**Figure 5:**
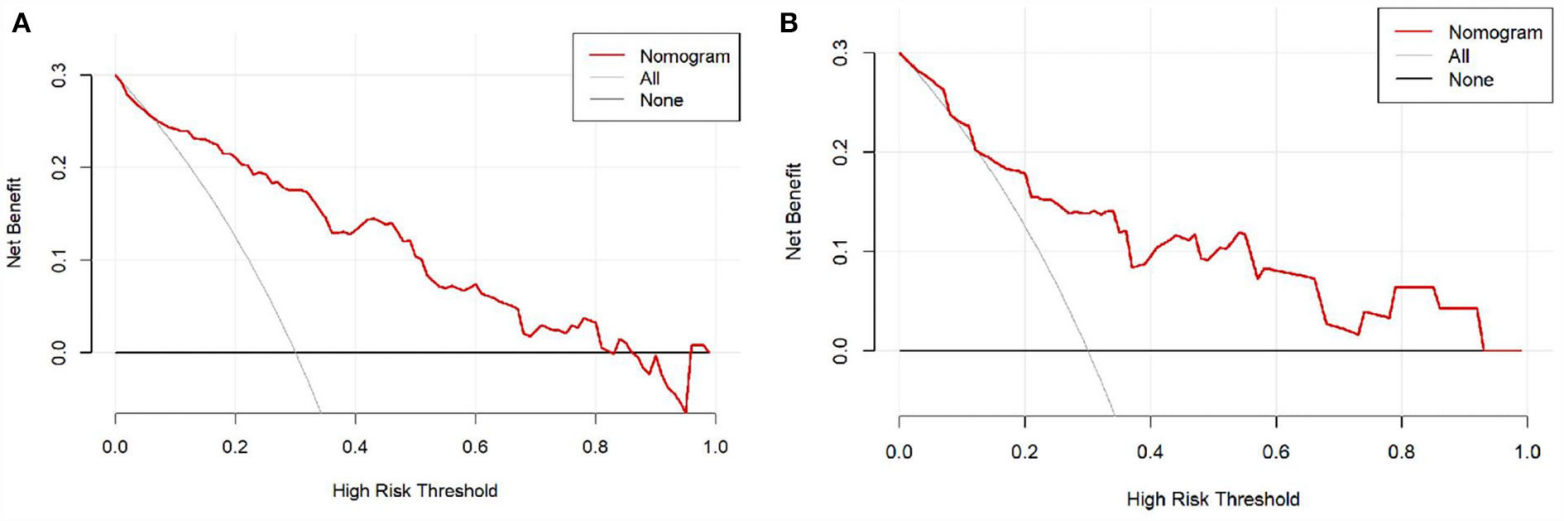
The decision curve analysis (DCA) of the nomogram in the training set and validation set. **(A)** DCA in the training set; **(B)** DCA in the validation set.

Moreover, the DCA was used to assess the clinical validity of the nomogram, which indicated the predictive nomogram to be clinically useful (Figure 5).

### The association between the serum SAA and the probability of PSCI, the serum SAA, and the MMSE score

**Figure 6:**
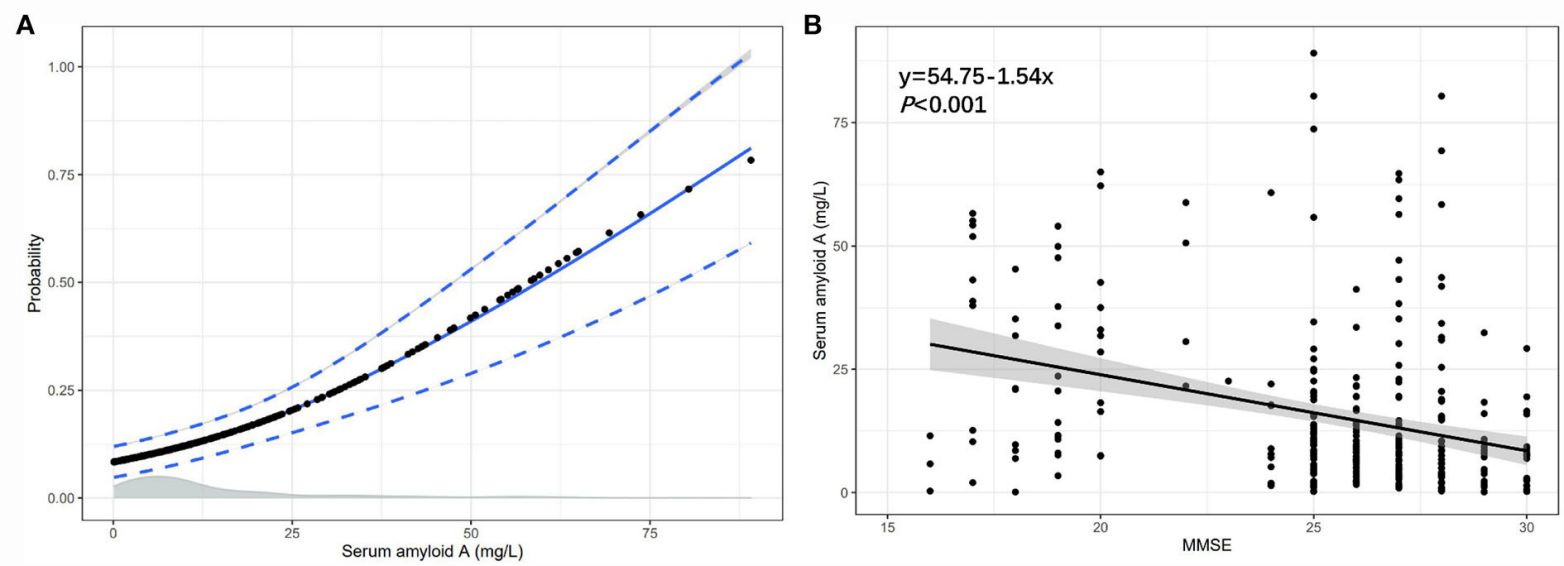
The association between the serum SAA and the probability of PSCI, the serum SAA, and the MMSE score. **(A)** The association of SAA levels with the probability of PSCI in patients with lacunar infarction; **(B)** The association between SAA levels and MMSE score.

We found a positive correlation between the serum SAA and the probability of PSCI, in which the predicted probabilities for PSCI were more than 50% after 58 mg/L of serum amyloid A (Figure 6A). Besides, linear regression analysis showed that the level of serum SAA was negatively associated with the MMSE score (regression equation: *y* = 54.75–1.54*x, P* < 0.001; Figure 6B).

## Discussion

Post-stroke cognitive impairment is a clinical syndrome of cognitive impairment that occurs after an ischemic stroke (18). Approximately 37.3% of respondents developed PSCI in a retrospective study of 209 patients with mild ischemic stroke (11). Therefore, it is important to assess PSCI early and conveniently. In this study, we investigated the association of SAA concentrations with the risk of cognitive impairment after lacunar infarction. Several pivotal results were found in this analysis. First, the increased SAA levels were significantly associated with a higher risk of PSCI. Second, we proved that several traditional risk factors, such as age, diabetes mellitus, white blood count, and cystatin C, were independent risk predictors of PSCI in patients with lacunar infarction. Third, we constructed a nomogram model based on SSA that can predict PSCI effectively.

Each brain's morphology is unique, and aging changes brain morphology in both healthy and pathological conditions (26). Heart failure, atrial fibrillation, and renal insufficiency may contribute to acute stroke with increasing age, especially in patients over 85 years of age (27). Overton's research found that the older age groups had more possibility of having cognitive impairment (28). Morley's study also came to the data that nearly 40% of the persons older than 65 years had mild cognitive impairment in the United States (29). In our study, we found a positive association between age and the occurrence of cognitive impairment after lacunar infarction. The key point of brain aging is the cellular senescence of neurons and microglia (30). Evidence proved that since the age of 40 years, about 5% of neuron cells are destroyed every 10 years, which directly leads to a decrease in brain volume (29). In addition, as an important component of immunity for the central nervous system, microglia plays an indispensable role to maintain tissue homeostasis (31). Since microglia are found around lesions in neurodegenerative diseases such as Alzheimer's disease, Parkinson's disease, and multiple sclerosis, the release of inflammatory factors mediated by microglia is thought to be the key to the onset or progression of neurodegenerative diseases (32).

Diabetes mellitus is a kind of noninfectious and multiple organs affected chronic disease (33). Liccini's research concluded that 20% of patients aged between 50 and 65 years were diagnosed with diabetes mellitus who had cognitive impairment, and the situation may be more severe in diabetes mellitus who had metabolic syndrome (34–36). Van Sloten et al. revealed that diabetes-related microvascular dysfunction affected the exchange of gases, nutrients, proteins, and metabolites in the human body environment (37), which was a key factor in the occurrence of cognitive impairment (38). In addition, the health of people with diabetes could be negatively affected due to cognitive impairment. Therefore, we need to face up to the fact that stroke patients with diabetes have more prevalence of cognitive impairment (39), and clinicians should pay more attention to the cognitive abilities of diabetes patients.

Inflammatory responses are closely related to ischemic stroke; it could promote the development of ischemic injury and neuronal death after stroke (40, 41). The higher white cell counts within the normal range were associated with cognitive impairment in older adults (42, 43). Studies based on population have confirmed the relationship between inflammation and cognitive impairment, that is, the inflammatory states can negatively impact cognitive function (44, 45). Furthermore, the animal experiment revealed that white blood cells promoted the immune system to degenerate brain tissue in stroke animal models (46). In our study, we confirmed that white blood count played an important role in predicting cognitive impairment after lacunar infarction. This suggested that neuronal inflammation prevention may reduce cognitive impairment and improve neurological outcomes in stroke patients.

Cystatin C is an endogenous cysteine proteinase inhibitor that exists nearly in all human cells and body fluids; it belongs to the type 2 cystatin superfamily (47, 48). Sarnak's research showed that higher levels of cystatin C were associated with cognitive impairment (49). Meanwhile, the higher serum cystatin C was an independent risk factor for PSCI in patients with acute mild ischemic stroke (50), which can provide early prediction of cognitive decline in the elderly (51). This is consistent with the conclusion of our study. Cognitive impairment could have a negative impact on the daily life of patients; therefore, reducing the level of serum cystatin C may provide a new treatment for the prevention of PSCI, and it is of great significance to timely predict the occurrence of cognitive impairment (52).

Serum amyloid A is a protein secreted by hepatocytes (53). The synthesis of SAA is associated with inflammatory cytokines, which can rise rapidly when infection and inflammation occur (18, 54). It is widely used as a follow-up marker for diagnosis, prognosis, or treatment of disease (55, 56). SAA has been recognized as being associated with cognitive impairment (57). Xu's research found the relationship between cognitive function and SAA levels in patients with vascular dementia and investigated the higher levels of SAA in patients with vascular dementia (58, 59). The elevation of SAA exacerbates neuroinflammation and changes the morphology of microglia to increase their activity, eventually leading to brain damage and memory loss (54, 60, 61). Therefore, for patients with lacunar infarction with elevated SAA, it is necessary for clinicians and healthcare organizations to take preventive actions against cognitive impairment that may occur in the future.

The nomogram based on the five variables would improve the predictive ability for PSCI in lacunar infarction patients. Compared with five independent risk factors, the nomogram exhibited good discrimination ability by the ROC analysis. In addition, DCA was applied in the training set and validation set, which confirmed the net benefit based on the threshold probability.

There were some limitations in this study. First, the study detected only the serum SAA levels within 24 h of admission, but did not examine the serum SAA levels before the stroke and within 3 months of discharge dynamically. Second, the patients did not perform the cognitive function assessment during admission, although patients with neurological disease and dementia were excluded. Third, the independent variables included in the study lack the relevant indicators of magnetic resonance imaging (cerebral atrophy and gray matter lesions), genetic risk factors, and environmental risk factors. Finally, there might be some bias in the selection of patients, because this study enrolled mild stroke patients with NHISS <3 only. Therefore, these issues need further exploration in the future prospective external studies.

## Conclusion

This study revealed the association of SAA level with PSCI, which was an independent risk factor to predict cognitive impairment in lacunar infarction patients. In addition, this study constructed the nomogram to predict PSCI based on the five independent risk factors, which has proven clinical utility and is useful for PSCI risk decision-making in patients with lacunar infarction undergoing clinical assessment.

## Data availability statement

The original contributions presented in the study are included in the article/supplementary material, further inquiries can be directed to the corresponding author.

## Ethics statement

The studies involving human participants were reviewed and approved by the Institutional Review Board of the Second Affiliated Hospital of Wannan Medical College. The patients/participants provided their written informed consent to participate in this study. Written informed consent was obtained from the individual(s) for the publication of any potentially identifiable images or data included in this article.

## Author contributions

SY and LX designed this study and provided the funding support. SY and HP drafted the first manuscript and analyzed the data. JX, WL, and BW took part in the sample collection and acquired the data. LX and BW followed up with the patient. LX reviewed and edited the manuscript. All authors have read and approved the final manuscript.

## Funding

This study was financially supported by the Key Research Fund Project of Wannan Medical College (WK2021ZF24 and WK2021ZF25) and the Summit plan of the Second Affiliated Hospital of Wannan Medical College (DFJH2022007).

## Conflict of interest

The authors declare that the research was conducted in the absence of any commercial or financial relationships that could be construed as a potential conflict of interest.

## Publisher's note

All claims expressed in this article are solely those of the authors and do not necessarily represent those of their affiliated organizations, or those of the publisher, the editors and the reviewers. Any product that may be evaluated in this article, or claim that may be made by its manufacturer, is not guaranteed or endorsed by the publisher.
